# A review of randomized controlled trials comparing the effectiveness of hand held computers with paper methods for data collection

**DOI:** 10.1186/1472-6947-6-23

**Published:** 2006-05-31

**Authors:** Shannon J Lane, Nancy M Heddle, Emmy Arnold, Irwin Walker

**Affiliations:** 1 Department of Medicine, McMaster University, Hamilton, Canada; 2 Canadian Blood Services, Hamilton, Canada

## Abstract

**Background:**

Handheld computers are increasingly favoured over paper and pencil methods to capture data in clinical research.

**Methods:**

This study systematically identified and reviewed randomized controlled trials (RCTs) that compared the two methods for self-recording and reporting data, and where at least one of the following outcomes was assessed: data accuracy; timeliness of data capture; and adherence to protocols for data collection.

**Results:**

A comprehensive key word search of NLM Gateway's database yielded 9 studies fitting the criteria for inclusion. Data extraction was performed and checked by two of the authors. None of the studies included all outcomes. The results overall, favor handheld computers over paper and pencil for data collection among study participants but the data are not uniform for the different outcomes. Handheld computers appear superior in timeliness of receipt and data handling (four of four studies) and are preferred by most subjects (three of four studies). On the other hand, only one of the trials adequately compared adherence to instructions for recording and submission of data (handheld computers were superior), and comparisons of accuracy were inconsistent between five studies.

**Conclusion:**

Handhelds are an effective alternative to paper and pencil modes of data collection; they are faster and were preferred by most users.

## Background

The use of portable handheld computer technology in the field of health care and clinical research is on the rise, with a corresponding increase in publications. A search of Medline, using the MeSH term "computers, handheld" and text words "handheld computer(s)" combined as "or", revealed a steady increase in the yearly number of publications from none in 1995 to 209 publications in 2003.

The use of handheld computers has been described in a variety of subjects and clinical settings, including: analgesic headache treatments[1]; pain research[2]; bipolar disorder research[3]; asthma research[4,5]; tobacco use research[6]; orthopedic research[7]; urinary incontinence research[8,9]; smoking cessation research [10,11]; brain injury research [12,13]; menstrual symptom research[14]; field data collection research[15,16]; diabetes research[17]; eating disorder research[18]; respiratory care research[19]; blood donor research[20]; and adolescent anxiety research[21]. Many of these studies have suggested that handheld technology has several advantages over traditional paper and pencil modes of data capture including but not limited to: data accuracy; timeliness of data capture; and adherence to protocols for data collection.

There are a number of published reviews on the use of handheld computers in the health care setting that focus on design aspects of the handheld device, advantages compared with paper methods, and the clinical applications in which they have been used [22-30]. These reviews provide the reader with much insight but they have limitations when comparing the actual performance of handheld computers with paper methods. Most of the literature is descriptive rather than comparative, focusing on the technology, the methods and/or the experience. When comparing the effectiveness of two methods, descriptive studies have limitations as they lack a comparative control group, and they are prone to publication bias and subject selection bias [31].

Despite the proliferation of literature describing the applications of handheld computers, the number of evidence based publications addressing the efficacy of this technology compared with the traditional method remains few. The purpose of this manuscript is to summarize the literature on randomized controlled trials focusing on the use of handheld computers compared to traditional paper and pencil methods, where at least one of the following outcomes was assessed: data accuracy; timeliness; adherence to protocols; and/or patient preference.

## Methods

### Literature search

NLM Gateway, a single interface that searches in "multiple retrieval systems", including MEDLINE^®^/PubMed^®^, was searched using the following text words, both separately, and combined with "OR": "palm top computer," "PDA," "personal digital assistant," "pocket computer," "electronic diary," "diary keeping," "diary keeping methods," "electronic forms and data collection," "microcomputer," "palm pilot," "handheld computer," and the MeSH headings "data collection/*instrumentation" and "computers, handheld". The search was performed during the period May 1st 2003 until June, 2005 and was restricted to publications in English. Two reviewers independently reviewed the citations and abstracts of all articles retrieved from the search (I.W. and S.L.) according to the inclusion criteria. Studies that met the following inclusion criteria were included in this review: study design was a randomized controlled trial (RCT); the study compared the use of handheld computer to paper and pencil as data collection devices; and at least one of the following outcomes was assessed – user preference, data accuracy, adherence, and timeliness. All potentially relevant articles were retrieved and reviewed. The bibliographies and reference lists of these documents were reviewed by one researcher (S.L.) to identify other potentially relevant articles that fit the inclusion criteria. Two reviewers independently classified the potentially relevant articles and then met to discuss discrepancies in classification. Articles in disagreement were discussed using consensus until both reviewers agreed upon the classifications. Data were extracted from each of the eligible studies by one of the authors (SL), under the following headings: study purpose; study design; duration of follow up; location; patient population; number of subjects; instrument and mode of entry; and outcome measures. Duration of follow up was considered important because preferences for new technologies might conceivably lack durability, initial enthusiasm and 'halo' effects clouding other issues of acceptability. The accuracy of the data extracted was confirmed by two of the authors (IW, and NH).

## Results

There were a total of 201 potentially relevant studies identified by the search strategy. After a review of the titles and abstracts 141 articles were retrieved of which 9 met the inclusion criterion for review in this study [32-40]. Table 1 provides a summary of the information extracted from each of the eligible RCTs included in this review.

**Table 1:** Summary of randomized controlled trials comparing handheld computers to paper and pen.

**Study**	**Purpose**	**Design**	**Duration of Follow Up**	**Location**	**Patient Population**	**Number of Subjects**	**Instrument and Mode of Entry**	**Outcome Measures**
Quinn P et al. 2003 ^33^	To assess the effectiveness of a portable electronic diary as a data collection device for symptoms of an overactive bladder (OAB)	Randomized crossover study	7 days/arm, 14 days total.	Patient's residence	Patients with a diagnosis of over-active bladder.	35 patients were recruited, 2 were excluded post randomization.	Intervention: • Customized version of MiniDoc • Daily diary • Visual Analogue Scale (VAS) Control: • Paper diary • Visual Analogue Scale (VAS)	Effectiveness of the electronic diary Acceptability to patients
Jamison RN et al. 2002 ^34^	To compare the e-VAS (electronic) with p-VAS (paper) for cognitive and sensory stimuli.	Single centre randomized crossover study	Data collected in a 1 hour session	Institution	Healthy volunteers	24 subjects	Intervention: • Palm Pilot IIIxe • Visual Analogue Scale (VAS) Control: • Paper Visual Analogue Scale (VAS)	Validity and equivalence of methods
Lal SO et al. 2000 ^35^	To determine whether electronic data collection and downloading to a personal computer spreadsheet is faster and more accurate than written data.	Randomized crossover design	Chart data collected within a 96 hour window period	Shriners Burns Hospital	Medical student volunteering for data collection	3 medical students retrieving data from 110 medical charts	Intervention: • 3Com Palm IIIx • Data entry into an Excel spreadsheet Control: • Paper duplicate of Excel spreadsheet.	Speed/Time Accuracy as measured by error incidence
McBride JS et al. 1999 ^36^	To examine how data can be collected at point of care. Comparison of electronic and paper versions of a standard quality survey.	Randomized design	Data collected in one session	Wake Forest PhysiciansOrthopedics Department Clinics	Patients visiting an orthopedic clinic	349 patients	Intervention: • Mini Doc, a portable electronic data capturing device. Control: • Paper and pencil survey form	Accuracy Acceptibility to patients Internal consistency reliability
Stratton RJ et al. 1998 ^37^	To assess an electronic visual analogue scale with a paper method for appetite rating To examine test-retest reliability.	Randomized crossover study design	4 day study Test-retest over 2 additional days	Subject's residence	Healthy free-living volunteers	12 volunteers participated in comparison study, 13 participated in preference study	Intervention: • Apple Newton Message Pad • visual analogue scale questionnaire Control: • Paper and pencil • visual analogue scale questionnaire	Comparability of methods (Equivalence) Patient preferences
Tiplady B et al. 1997 (study 1) ^38^	To assess the suitability of PDAs compared to paper diaries, for daily collection of data on lung function.	Randomized two period crossover design	1 month/arm	Patient's residence	Out-patients with chronic obstructive airways disease	22 patients	Intervention: • Apple Message Pad • Daily diary Control: • Paper and pencil • Daily diary	Comparability of methods, re: data quality (missing and problematic data)
Tiplady B et al. 1997 (study 2) ^38^	To assess the suitability of electronic diary for home use, transmitting respirology data.	Observational study	Completed electronic diary for 1 month	Patient's residence	Patients with chronic airways disease.	37 patients	Intervention: • Apple Message Pad. • Daily diary	Patient preferences Ease of use
Drummond HE et al. 1995 ^39^	To compare the responses obtained from a quality of life (QOL) questionnaires using electronic (PDA) and conventional (paper).	Randomized, open, two period crossover	3 office visits; 1 for training, 1 to complete each arm.	Institution	Patients attending a gastrointestinal clinic as outpatients	46 patients	Intervention: • Apple Newton Message Pad. • Quality of Life questionnaire (QOL) Control: • Paper and pencil	Comparability of methods, looking at missing and problematic data Patient preferences
Rivellesse AA et al. 1991 ^40^	To evaluate an electronic (Food-Meter) method for recording 7-day food intake.	Randomized cross-over design repeated once.	4 weeks	• patient's residence	Insulin-dependent diabetic patients (IDDM)	21	Intervention: • "Food-Meter" (Miles, Elkhart, IN) • Daily diary Control: • Paper and pencil • Daily diary	Agreement between methods
Walker I et al. 2004 ^32^	To compare handheld computers and paper diaries for recording intravenous infusions of hemophilic clotting factor concentrates.	Randomized controlled trial, parallel design.	6 months	• patients' residence	Patients with hemophilia	41	Intervention: • Palm III with bar code reader Control: • Touch-sensitive paper diary.	Compliance Timeliness Accuracy Preference

Topics addressed by the nine randomized studies were: Symptoms in patients with overactive bladder[33], appreciation of pain by volunteers[34], collection of chart data by medical students[35], symptoms of patients in an orthopedic clinic[36], rating of appetite by volunteers[37], respiratory data in patients with lung diseases[38], quality of life in patients with gastrointestinal disease[39], food intake by patients with diabetes[40] and factor concentrate use by patients with hemophilia[32]. Three of the studies used a parallel RCT design[32,36,39]; whereas, the remaining studies used a randomized crossover design[33-35,37,38,40]. The duration of the follow-up in the studies varied in length but in all cases would be considered short term i.e. same-day experiments[34,36], follow up lasting two to three days [35,37,39]; follow up over a one week period [33]; and two other studies where follow-up lasted one month per arm [38,40]. One study was carried out over six months[32]. In five of the studies the data collection occurred at the patient's residence[32,33,37,38,40], while the other four studies were conducted at institutions (i.e.: clinic, physician's office, hospital) [34-36,39].

The electronic forms of data entry were variable, utilizing push button technology[33,36], or touch sensitive screens using a stylus pen [34,37-39]. In one study data were entered via bar codes and an optical reading system[40], while the mode of entry for one study was not clearly specified [35]. In the remaining study [32] data was entered into a handheld computer by a combination of an integrated bar code reader and stylus pen.

Handheld computers and paper and pencil technologies were compared using a variety of applications. In three of the studies subjects were asked to rate some kind of subjective symptom or experience using visual analogue scales (VAS) [33,34,37]; whereas in a study of patients with asthma daily symptoms were recorded using custom software[38]. In two of the studies, questionnaires regarding quality of life [39]and quality of care [36] were administered in paper and electronic forms. Three studies examined the abilities of both devices to record objective data such as burn variables[35], daily food intake [40]and intravenous infusions of hemophilic clotting factor concentrates[32].

In three of the studies healthy volunteers/medical students participated to evaluate the two methods of data collection: electronic using a handheld or paper based manual collection [34,35,37]. The remaining six studies involved a variety of patient populations including patients with: bladder disorders[33], orthopedic problems[36], chronic obstructive airway disease[38], gastrointestinal problems,[39] diabetes [40] and hemophilia [32]. Summaries of the different studies are given in Table 1.

All, but one of the studies in this review have explored more than one outcome measure[40]. Three studies evaluated the *acceptability *of handheld computers to patients [32,33,36]. In one of these studies acceptability was determined by comparing percentages of positive features reported for each device [36]; the second study measured acceptability using an ease-of-use scale and a face-to-face interview questionnaire to elicit subject's opinions [33]; in the third study [32], acceptability was not assessed but ten (50%) of the patients who had used both paper diary and handheld computer methods subsequently took part in a subsequent qualitative study [41]. Three studies looked at the *comparability *of handheld computers to paper and pencil devices: two of the studies compared missing and problematic data points [38,39]; and one study compared results obtained using both paper and electronic visual analogue scales [37]. Two studies explored the *equivalence *of data obtained by electronic and paper and pencil methods, one used VAS [34], and the other explored differences between group scale scores using the Wilcoxon test [36]. Two studies assessed the *validity *of electronic modes of data capture: one study attempting to validate an electronic VAS [34]; the other study measuring *agreement *between conventional and electronic devices in order to validate an innovative device used for data capture pertaining to food intake [40]. The *speed *and *accuracy *of both methods were measured in one study [35], as was *effectiveness *[33] in another. The final study [32] compared adherence to a set schedule for submission of data, *accuracy *of data, and *lag time *between data entry in the home and receipt of data at the clinic. Reminder phone calls were made according to a strict protocol.

### Data accuracy

The findings related to an outcome of accuracy are summarized in Table 2. Six out of nine studies [32-36,38]compared data accuracy obtained by handheld technology to that of paper and pencil devices. Measurements of accuracy differed between studies. In two trials [34,36] data accuracy was measured by comparing the equivalence of responses obtained using both paper and pencil and hand-held microcomputer data collection modes. The authors found a similar degree of accuracy between results recorded on a paper and pencil visual analogue scale and VAS administered via a handheld microcomputer. Similarly, a comparison of paper and pencil and handheld microcomputer versions of the PRESS, GANEY™ outpatient survey questionnaire determined that both modes of data capture produced comparable results. In two other studies, data quality/accuracy were measured by comparing the percentages of errors obtained by each device [33,35]. Both studies found that data collected electronically were more accurate and contained fewer errors than data captured manually with paper and pencil. In one case, the paper and pencil mode of data collection produced a 6.7% error frequency, compared with a significantly lower 2.8% error frequency obtained by patients using the microcomputer technology [35]. The other study [32] detected errors in 80% of the paper diary data, but found the error rates of electronic diaries to be low, hypothesizing that the prompts and structures of questions and responses guide the user towards greater accuracy. Another study [38] measured data accuracy by comparing the proportions of 'missed' and/or 'problematic data' points between the two methods; detecting a higher proportion of missing and/or problematic data in the PDA diaries. The final study [32] compared the number of medication vials unaccounted for and also the proportion of individuals having errors in the number of vials not accounted for; the error rate was similar for the two methods.

**Table 2:** Summary of the results of data accuracy assessed in six randomized controlled trials

**Study**	**Results related to data Accuracy**		**Definitions**
		
	**Hand Held Computers**	**Paper**	
Tiplady B et al. 1997 ^38^	Missing data 8.91% Problematic data** 5.64%	Missing data 0.16% Problematic data** 0.24 %	Accuracy was defined by comparing missing and problematic data between the two methods. **Problematic data points defined as those requiring some intervention or editing.
McBride JS et al. 1999 ^36^	No difference in missing item responses between PDA and paper in 4/5* subscales, (p < 0.05).	No differences in missing item responses between PDA and paper in 4/5* subscales, (p < 0.05).	Defined as a comparison of missing item responses between the two methods.
Lal SO et al. 2000 ^35^	2.8% error frequency.	6.7% error frequency	Data fields analyzed for frequency of error were gender, race, date of birth, date of burn, date of admittance to hospital, and burn type. Accuracy determined by comparing these fields with original medical record.
Jamison RN et al. 2002 ^34^	Of 503 paired verbal stimuli in 24 subjects the correlation between paper and PDA ratings was r = .97 (range 0.95–0.98), for sensory stimuli r = 0.86 (range 0.81–0.92). Correlation between group electronic VAS and paper VAS ratings to common verbal stimuli r ^2 ^= 0.997, for the common sensory stimuli group correlation was r ^2 ^= 0.99.		Defined as the degree of correlation between the two methods of rating.
Quinn P et al. 2003 ^33^	Errors not possible in electronic diaries due to prompt and format of questions and responses.	Errors "detected" in 80% of paper diaries	Errors defined as incomplete times, inconsistent timing of events, incomplete events, incorrect completion of VAS.
Walker I et al. 2004 ^32^	3 vials/patient not accounted for. 15 patients with errors.	5 vials/patient not accounted for (P = 0.45). 13 patients with errors (P = 1.00).	Both the number of vials/patient not accounted for in each group and the number of patients in each group with errors.

*The subscale in which differences in missing item responses were found was the 1^st ^response choice on PDA, authors suggest this could be attributed to learning effect.

### Timeliness

Table 3 provides a summary of findings related to the timeliness of data collection. Four out of the nine RCTs report outcomes comparing the timeliness of electronic portable microcomputer technology to paper and pencil data collection devices [32,35,38,40]. One of the three trials [35] directly measured data entry time between electronic and paper and pencil devices. In this study [32] the use of electronic instruments reduced data entry and transfer time by 23%. A second study [32] compared the interval between the times of intravenous infusions and the receipt of data; the interval from those using handheld computers was greatly reduced (0.25 vs. 25 days, p < 0.0001). The remaining two studies [38,40] reported that electronic instruments reduced the time required for data handling and transfer but did not provide time estimates.

**Table 3:** Summary of the findings related to timelines in the four studies reporting this outcome.

**Study**	**Timeliness**		**Conclusions**
		
	**Handheld Computers**	**Paper and Pencil**	
Rivellese AA et al.1991 ^40^	1 minutes/week to transfer data into computer with 'Food-Meter' cable hook up.	> 30 minutes/week with conventional method. Specific data not provided.	No conclusions were made
Tiplady B et al.1997 ^38^	Authors claim electronic diary automation reduced total data handling time by over 80%; specific data not provided.	Specific data not provided	No conclusions were made
Lal SO et al. 2000 ^35^	Average PDA data collection and hook up time was 50 minutes for every group of 10 patients entered	Manual collection with computer entry method was 65 minutes for each group of 10 patients. Total time decreased by 23%	Handheld computers decreased total time by 23%
Walker I et al. 2004 ^32^	0.25 days from intravenous infusion to receipt of data. Reminder phone calls 1/patient	25 days from intravenous infusion to receipt of data. Reminder phone calls 5/patient	Major statistical and clinical significance.

### Adherence

Three of the nine studies addressed the issue of adherence [32,33,38] but only one provided objective data [32]. In this latter study a schedule for submission of data was provided prospectively to the patients; adherence by users of handheld computers was 86% versus 48% for paper diary users (P < 0.0001) despite an increased number of reminder phone calls to users of paper diaries compared to the users of handheld computers (5 versus 1, P < 0.0001). Neither of the other two studies [33,38] made direct measurements of adherence between electronic and paper/pencil modes of data collection. In one trial [33] adherence was evaluated for electronic data capture but not for paper and pencil data collection. In the same study where patients were instructed to complete diaries daily, recording events as soon as possible after occurrence, 73% of the cases entered data into the diaries within two hours of the event occurring. In one study, where adherence was not measured [38] the authors report unconfirmed suspicions of retrospective data entry in paper diaries, which relates specifically to the issue of adherence.

### Patient preference

Table 4 provides a summary of the data related to patient preferences. Four of the nine RCTs evaluated patient preference as a secondary outcome measure, using patient surveys [32,37-39] while ten of the twenty-two patients in a fourth study who had been randomized to handheld computers but who had previously used paper diaries took part in a subsequently published qualitative study [41]. Three out of four trials found that subjects preferred portable handheld devices to paper and pencil [32,38,39], while in the fourth study a small majority of subjects preferred a paper and pencil questionnaire [37]. In total, 54/91 (59%) of subjects favored the used of the PDA, 17/91 subjects (19%) favored paper methods of data capture. Twenty out of 91 subjects, (22%) indicated "no preference".

**Table 4:** Summary of RCTs assessing patient preference

**Study (Author, Year)**	**Proportion of Patients with a Preference (# [%])**		
	
	**PDA**	**Paper**	**No Preference**
Drummond HE et al., 1995 ^39^	26/46 (57)	6/46 (13)	14/46 (30)
Tiplady B et al., 1997 ^38^	13/22 (59)	4/22 (18)	5/22 (23)
Stratton RJ et al., 1998 ^37^	5/13 (38)	7/13 (54)	1/13 (8)
Walker I et al.,2004 Arnold E et al., 2005 ^32^ (Follow-up qualitative study)	10/10 (100)	0/10 (0)	0/10 (0)

## Discussion

Despite the numerous investigations describing the potential advantages that hand-held computers offer in research and health care settings compared to traditional paper data collection methods [2,4,6,8,9,15,16,42,43] only a handful of these studies adhered to the ideal, experimental method for the evaluation of effectiveness – the RCT (Randomized Controlled Trial)[44]. However, we were able to identify nine studies that comparatively evaluated handheld computers and pencil and paper methods using an RCT crossover or parallel design. The results of this review suggest that handheld computers have the potential not only to overcome some of the limitations of conventional paper and pencil devices but to supersede them, particularly with respect to improving timeliness of data handling. In addition, the preference by research subjects for handheld computers could result in improved adherence to data collection protocols for long-term studies, as evidenced by the markedly improved adherence and patient preference for the handheld computer group in the study having the longest observation period [32].

The importance of data quality from investigator's and sponsors' perspectives has been highlighted elsewhere as the most important issue in considering new data capture technologies [45]. On the other hand an increased accuracy of data entry cannot be assumed for either method. In this review only two of the six studies found handheld computers to be more accurate, in three studies accuracy was similar and in one study the paper method was more accurate. Data entry with handheld computers can in some cases be made relatively foolproof by carefully structuring the questions to allow only determinate types of responses and by the use of prompts to ensure that questions are followed in sequence and cannot be skipped. However, handheld computers will not result in greater accuracy of data where the performance of the paper method is already at a high level or where the source of error is in data collection rather than in data entry or transmission.

Recently, the utility and validity of paper and pencil diaries has been called into question by the findings of Stone et al's [46] suggesting high levels of faked compliance in paper diaries. The same study also demonstrates the capacity of handheld computers to improve and provide objective measures of adherence to protocols by time and date stamping data entries [47]. The authors of one study in this review made similar speculations about retrospective data entry after finding high rates of missing data using handheld computers (designed not to permit retrospective entry) but low rates of missing data in paper diaries [38]. Despite Stone et al's invention and validation of a paper diary equipped with a photo sensor and capable of detecting opening and closing of the diary [47] only one of the studies in this review evaluated direct comparisons of adherence between methods[32]. This latter study evaluated adherence to a set schedule for data transmission rather than evaluating adherence to timely data entry. Future research should be directed towards the endeavor of making direct comparisons of adherence between methods as part of the larger project of validating handhelds for data capture among patient populations.

Differences between the handheld and paper and pencil instruments in data entry, in data handling and transfer times, were seldom evaluated in these studies, yet, theoretically handheld computers offer enormous temporal and financial benefits that deserve further exploration in clinical research. Typically in research vast quantities of data must be collected, organized, and transcribed by key entry into a file using computer software. Often this is done twice (double-data entry) to ensure accuracy and reduce the potential for human error. Using a handheld computer for data collection can eliminate these processes, which are costly and time consuming, resulting in a superior method to conventional paper and pencil devices.

As care and treatment strategies become increasingly patient centered, patient preferences are taken into greater consideration. Preference is defined as the level of desirability that a person associates with a particular health state, treatment process or level of participation [48]. Accordingly, 4 of the 9 studies summarized in this review evaluated patient preferences for either electronic or paper and pencil data capture at the end of the studies. In three out of four of these studies a larger percentage of patients preferred handheld computers to paper and pencil for data capture (Table 4). These findings are important because research has shown that patient satisfaction can influence the efficacy of interventions and levels of compliance [48]; therefore it seems in the interests of the investigator to consider and incorporate patient preferences when choosing a data collection tool. A recent study from our centre has also shown that method of record keeping is an important factor contributing to adherence of reporting [41].

The analysis of these studies provides evidence that handheld computer devices are an effective technology for data collection in the health care setting and in health related research. When compared to paper and pencil methods of data recording the handheld computers appear to be faster and preferred by most patients. The accuracy of the data collected may be greater with handheld computers in some circumstances but not all and the definitions of accuracy varied between studies. Future studies in this area would benefit from a more standardized definition of accuracy to allow for inter-study comparisons of results. As well, additional information to assess adherence to data collection requirements would also be useful as part of the overall assessment of the usefulness of handheld computer technology for data collection in the health care setting.

Recently, a number of authors have stressed the limitations of randomized trials in assessing the role of computer systems, indicating that questions such as how and why computer systems are used and explanations of various phenomena are best answered by studies based on qualitative, technical, psychological and other methods [49-51]. None of these authors are however suggesting that randomized trials be abandoned, and in fact are suggesting an integrated approach involving both quantitative and qualitative methods. Our opinion is that the randomized trial is best suited for comparing the ultimate performance of two or more methods in their actual clinical settings. Questions of generalizability to situations outside the actual test conditions will arise, but this is well understood. We suggest that the emphasis, though not the only role, of other methods is in the explanation of observed phenomena and in the actual development of systems. In deciding on a method of data collection, researchers could reasonably consider that handheld computers will likely be acceptable to patients and have the potential to provide more rapid data handling. On the other hand, improved compliance and accuracy of data recording should not be assumed and may depend on the particular conditions of the study, the origin of these aspects may lie outside the nature of the recording device, such as in training, understanding and motivation.

## Conclusion

These studies illustrate many of the technical qualities of handheld computers described in previous reviews and are highlighted by their direct comparison with those of the paper standard when these methods are used side by side. Handheld computers can be programmed to provide determinate responses, date stamped to document times of data entry, restrict times of data entry, prevent retroactive data entry, limit 'look back' to previous data, prevent omissions of data entry, and can save considerable time and labor incurred in data handling. Handheld computers are well accepted, and are more likely than paper methods to be the choice of the user. The ultimate results with handheld computers have in most trials been similar to those of the paper method, particularly when the performance of the paper method is already high, and therefore improved accuracy cannot be assumed. The potential advantages of handheld computers lie in their technical advantages which should be carefully considered when designing the software programs to match the task.

## Competing interests

The author(s) declare that they have no competing interests.

## Authors' contributions

SL carried out the research and took the lead role in preparing the manuscript; NH was the academic supervisor of SL, assessed the accuracy of the search, reviewed the selected articles and provided major editing of the manuscript; EA provided SL with assistance in defining search criteria and methodology, and reviewed the manuscript and many of the articles; IW conceived the study, independently selected the articles and provided major editing. All authors provided approval to thesubmitted manuscript.

## Pre-publication history

The pre-publication history for this paper can be accessed here:


